# The cholesterol-lowering effect of unripe *Rubus coreanus* is associated with decreased oxidized LDL and apolipoprotein B levels in subjects with borderline-high cholesterol levels: a randomized controlled trial

**DOI:** 10.1186/s12944-020-01338-z

**Published:** 2020-07-09

**Authors:** Jung Min Cho, Jisuk Chae, Sa Rang Jeong, Min Jung Moon, Ki-Chan Ha, Sunoh Kim, Jong Ho Lee

**Affiliations:** 1 National Leading Research Laboratory of Clinical Nutrigenetics/Nutrigenomics, Department of Food and Nutrition, College of Human Ecology, Yonsei University, 50 Yonsei-ro, Seodaemun-gu, Seoul, 03722 South Korea; 2 Department of Food and Nutrition, Brain Korea 21 PLUS Project, College of Human Ecology, Yonsei University, Seoul, South Korea; 3 Healthcare Claims & Management Incorporation, Jeonju, Republic of Korea; 4 B&Tech Co., Ltd., R&D Center, Gwangju, 61239 South Korea

**Keywords:** *Rubus coreanus*, Unripe *Rubus coreanus*, *Rubus coreanus* Miquel, Cholesterol-lowering, Low-density lipoprotein, Apolipoprotein B, Oxidized LDL

## Abstract

**Background:**

*Rubus coreanus* (*R. coreanus*) possesses properties that may decrease cholesterol levels.

**Methods:**

The effects of unripe *R. coreanus* (uRC) consumption on low-density lipoprotein (LDL) and total cholesterol levels related to decreased circulating apolipoprotein (Apo) B and oxidized LDL levels were evaluated. This randomized, double-blind, placebo-controlled study included subjects with borderline-high cholesterol levels (between 200 and 239 mg/dL) who consumed one capsule daily containing 600 mg of freeze-dried uRC extract (*n* = 39) or the placebo (*n* = 38).

**Results:**

After 12 weeks, the uRC group showed reductions of 21.23 ± 4.36 mg/dL in total cholesterol levels (*P* = 0.007) and 15.61 ± 4.16 mg/dL in LDL cholesterol levels (*P* = 0.032). In addition, significantly greater reductions in Apo B levels were observed in the uRC group (− 3.48 ± 3.40 mg/dL), but Apo B levels were increased in the placebo group (6.21 ± 2.84 mg/dL; *P* = 0.032). Furthermore, a remarkably lower oxidized LDL level was detected in the uRC group (57.76 ± 2.07 U/L) than in the placebo group (66.09 ± 3.47 U/L) after 12 weeks of consumption (*P =* 0.044).

**Conclusions:**

Because of its cholesterol-lowering effect, uRC shows great promise as a therapeutic agent for subjects with borderline-high total blood cholesterol levels.

**Trial registration:**

ClinicalTrials.gov Identifier: NCT03649620 (8/28/2018, retrospectively registered).

## Introduction

High total and low-density lipoprotein (LDL) cholesterol are potentially important risk factors for hyperlipidemia [1–3]. Moreover, the presence of elevated blood levels of total cholesterol is considered a risk factor for cardiovascular disease (CVD) [4]. The American Heart Association defines a normal total cholesterol level as less than 200 mg/dL [5–7], whereas a total serum cholesterol level of 200 mg/dL to 239 mg/dL is “borderline-high” and a total cholesterol level greater than 240 mg/dL indicates an increased risk of heart disease. Borderline-high total cholesterol levels can precede dyslipidemia and severe CVD by several years or decades [8, 9]. High total cholesterol and LDL cholesterol levels are conditions that represent the onset of dyslipidemia [10] and are reported to be critical factors contributing to atherosclerosis, myocardial infarction, and stroke [11].

Many studies have investigated the effects of herbal/dietary supplements on lipid disorders. Sumac (*Rhus coriaria*) was shown to alter lipid profiles, including increasing apolipoprotein (Apo) A-I and high-density lipoprotein (HDL) levels and decreasing triglyceride (TG), total cholesterol and LDL cholesterol levels [12]. In addition, Damavandi et al. reported that fatty acids from cashew nut reduced the LDL/HDL cholesterol ratio in patients with type 2 diabetes [13]; in contrast, another review concluded that cashew nut consumption alone did not significantly alter lipid profiles, including levels of LDL or total cholesterol [14]. According to interventional studies, α-linolenic acid and plant omega-3 (*n*-3) fatty acids from flaxseed (*Linum usitatissimum*) possess cholesterol-lowering effects [15] and reduce CVD risk [16]. Additionally, polyphenolic natural products, such as curcuminoids, which are present in the dietary spice turmeric (*Curcuma longa*)*,* are also used as therapeutic agents for patients with hypercholesterolemia [17]. Thus, interventions using natural products might differentially affect the modulation of the lipid profile in humans.

*Rubus croreanus* Miquel (black raspberry, RC) is part of the Rosaceae family and grows in the Republic of Korea [18]. The extract of RC contains abundant levels of various natural components, such as ellagic acids, flavonoids, tannins, phenolic acids, organic acids, tyrosol, and resveratrol [19, 20]. These pharmaceutically active components exert antihyperlipidemic, anti-inflammatory and antiatherosclerotic effects [21–24]. In addition, the degree of fruit ripening affects the yield of inflammatory modulators [23]. In a previous study, ethanol extracts of unripe fruit exerted the greatest effect on reducing nitric oxide (NO), prostaglandin E2 (PGE2), and proinflammatory cytokine levels in lipopolysaccharide (LPS)-stimulated RAW264.7 murine macrophages. Moreover, high-performance liquid chromatography (HPLC) showed that ellagic acid, one of the physiologically active elements of unripe RC (uRC), was present at significantly greater levels in the unripe fruit than in the ripe fruit.

According to one clinical trial in humans, the use of black raspberry improves vascular endothelial function and significantly reduces the levels of total cholesterol and inflammatory cytokines in patients with metabolic syndrome [25]. However, the efficacy of uRC has mainly been explored in vitro or in animal models. Well-designed randomized controlled trials in human subjects are limited or nonexistent.

This study hypothesized that uRC consumption modulates the lipid profiles in subjects with borderline-high cholesterol levels. Thus, a randomized, double-blind, placebo-controlled trial (RCT) was designed to investigate total cholesterol and LDL cholesterol levels along with circulating Apo A1, Apo B, and oxidized LDL levels in 80 subjects following a 12-week treatment with a uRC supplement.

## Methods

### Study subjects

Study participants included 80 subjects with total blood cholesterol levels ranging from 200 to 239 mg/dL. Initially, 252 volunteers who responded to advertising by the Clinical Nutrigenetics/Nutrigenomics Laboratory at Yonsei University between April 2017 and March 2018 were screened. The eligibility criteria included adult male and female participants aged 20 years to 65 years. Upon enrollment, the study participants’ total cholesterol levels were measured again by analyzing fasting blood samples. Exclusion criteria included a high LDL cholesterol level ≥ 160 mg/dL, the use of lipid-lowering or antihypertension or anticoagulation drugs within the 6 months before participation, diabetes, or a history/presence of significant metabolic disease. Furthermore, subjects with history of chronic infection, liver disease, cardiovascular disease, kidney disease, gastrointestinal disease, or any other acute disease requiring treatment were excluded. The present study ultimately included 77 subjects, excluding three subjects who voluntarily dropped out, and no one withdrew or was excluded due to poor compliance less than 80%. Compliance was defined as “taking a single capsule containing 600 mg of the experimental supplement or placebo daily” and was calculated as a percentage using the following equation: (number of days the capsule was taken (N)/number of days of total follow-up (N)) × 100%. The study protocol was approved by the Institutional Review Board of Yonsei University, according to the Declaration of Helsinki (IRB No. 7001988–201,808-SB-303), and written informed consent was obtained from the subjects prior to participation.

### Study design, intervention and group size

A 12-week RCT was conducted with 80 subjects who had total cholesterol levels of 200–239 mg/dL; all subjects were divided into two groups, the uRC (test) group and the placebo (control) group. Over the 12-week testing period, the uRC group consumed one capsule (800 mg/capsule) containing 600 mg of freeze-dried uRC once a day (1 h before eating dinner). The subjects in the placebo group were provided the designated capsules that did not contain dried uRC. The test product pills, which contained the uRC extract mainly as a freeze-dried powder, and the placebo-product pills containing starch were identical in appearance and organoleptic properties. Randomization was performed using computer-generated block randomization (placebo:test = 1:1). Test products were supplied by B&Tech Co., Ltd. (Gwangju, Jeollabukdo, Rep. of Korea) as individual packets of tablets. The sample size was determined and calculated by referring to the cholesterol-lowering results obtained from another clinical trial that reported statistically significant results [26]. According to the superiority test used in that study, the change in the cholesterol level of the test group was 14.6 mg/dL (μt) and of the placebo group was 6.1 mg/dL (μc); the standard deviation of the cholesterol level was 13.6 mg/dL (σt and σc). The sample size was then designed based on a two-sample t-test power calculation with a type II error (β) of 0.2, a power of 0.8, and a level of significance (α) of 0.05. The calculation resulted in a minimum of 32 subjects per group (λ = 1), and the dropout rate was estimated to be 20%; thus, a sample size of 40 participants per group was selected to increase the statistical power.

### Subjects with borderline-high total cholesterol levels and indications of healthy functional food

**Fig. 1:**
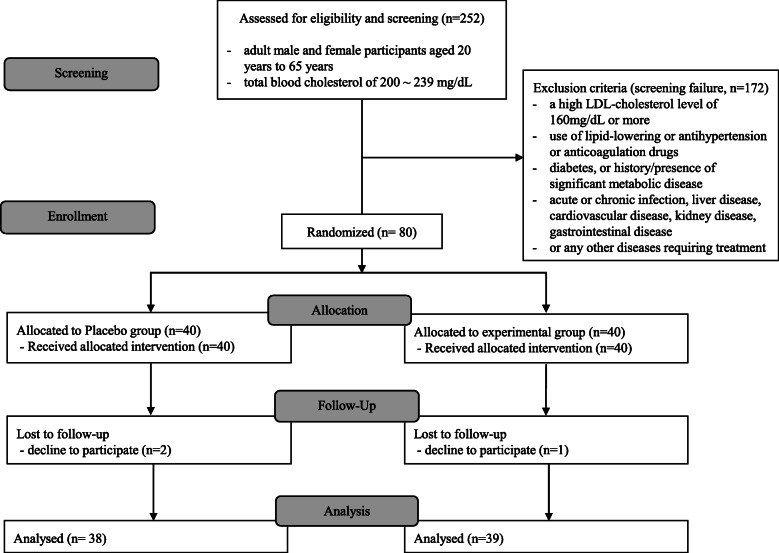
Flow diagram of participants

A flow diagram of the randomization of subjects and the inclusion and exclusion criteria is provided in Fig. 1. In the present study, the total cholesterol level (200–239 mg/dL) was used as the inclusion criterion, and subjects with LDL cholesterol levels ≥160 mg/dL were excluded. Although established international standards are not available for these subjects, the Adult Treatment Panel III of the National Cholesterol Education Program (NCEP) and the American Heart Association definitions of “borderline-high” total cholesterol levels ranging from 200 to 239 mg/dL and “high” total cholesterol levels > 240 mg/dL were used [27]. An LDL cholesterol level ≥ 160 mg/dL was defined as “high”. In addition, the current study was conducted to assess the effect of a cholesterol-lowering functional food (uRC) on a healthy subject or a subject in poor health in accordance with the guidelines of the Korea Food and Drug Administration (KFDA) [28]. This guideline emphasized that the subject should be in the borderline-high total cholesterol category between 200 and 239 mg/dL because a ‘food’ was used as the intervention rather than a drug. Therefore, this clinical trial should be conducted as an RCT designed to confirm the functional status of the human body, and the subjects should be healthy subjects or subjects in poor health [29].

A health functional food (HFF) is a nutritious product manufactured with ingredients or components that exert physiological effects. Unlike drugs, the functionality of HFFs is not to directly treat or prevent diseases, but to promote useful effects for health purposes by adjusting nutrients or physiological effects on the structure and function of the human body. Although the subjects’ total cholesterol levels were in the borderline-high range and were not an indication for drug treatment, a functional food can be used and was indicated for the participants in this study. Therefore, subjects with borderline-high total cholesterol levels were defined as proper subjects in this study.

### Information, manufacture and dosage of the test product

The uRC used in the present study was collected (May 2016) in Gochang County (Jeollabukdo, Rep. of Korea) and authenticated by Dr. Kim at B&Tech Co., Ltd. Recently, *R. coreanus*-specific markers in the test product were successfully validated using multiplex PCR markers based on chloroplast DNA [30]. The properties of the primers using the single nucleotide polymorphism (SNP) and insertion and deletion (INDEL)-based markers at *rp*l16 and *trn*G–*trn*S intragenic spacer regions confirmed that the test product was *R. coreanus.* Experimental products were manufactured according to Good Manufacturing Practice (GMP) by BCL Bio Co., Ltd. (Jincheon-gun, Chungcheongbuk-do, Korea) and examined in vivo and in vitro [31–33]. For dose-escalation experiments, the effects of the test extract were investigated by feeding C57BL/6 mice a high-cholesterol diet in a previous study conducted by the authors. The greatest decrease in total cholesterol, TG and LDL cholesterol levels occurred in the 300 mg/kg/day group and the smallest decrease was observed in the 50 mg/kg/day group, with significantly reduced glutamic oxaloacetic transaminase (GOT) levels, glutamic pyruvic transaminase (GPT) levels, and atherogenic index values [33]. In another study, the concentrations of 0.1, 0.3, 1, 3, 10, 30, and 100 *µ*g/mL of the test extract inhibited the lipid accumulation rates by 0.96 ± 2.83%, 2.43 ± 4.12%, 8.14 ± 2.0%, 9.52 ± 4.08%, 21.05 ± 2.63%, 37.11 ± 2.30%, and 56.28 ± 1.10%, respectively, in a dose-dependent manner [32].

The uRC extract was prepared using the technique established by Lee and Kim [34]. Briefly, uRC was extracted with 5% ethanol (95% ethanol, Daehan Ethanol Life, Seoul, Korea) at 100 °C for 4 h. The extract was then filtered, concentrated with a vacuum to evaporate the solvent, and freeze dried. The active formulation tablets (800 mg) each contained 600 mg of uRC. Placebo tablets (800 mg) were prepared using inactive ingredients that were identical to those in the active tablets by substituting the extract with 614 mg of starch. In this human clinical trial, 600 mg/day of the experimental product were used. According to previous in vivo and in vitro studies, the animal dose (mouse) was 100 mg/kg, and the dosage was calculated as follows: Human Equivalent Doses (mg/kg) = Animal dose (100 mg/kg) × (Animal kg/Human kg (3/27)) = 8.11 mg/kg. The average human body weight was estimated as 70 to 75 kg; thus, 8.11 mg/kg × (70 to 75 kg) = 567.7 to 608.3 mg/day were ultimately administered.

### Anthropometric parameters, blood pressure and blood collection

All anthropometric parameters were measured in subjects wearing lightweight clothes and no shoes. Body weights and fat percentages were recorded via the bioelectrical impedance method using a digital scale (UM0703581; Tanita, Tokyo, Japan). Heights (GL-150; G-tech International, Uijeongbu, Korea) were measured, and then the body mass index (BMI; kilograms per square meter (kg/m^2^)) was calculated. Systolic and diastolic blood pressure (BP) were measured on the left arm using an automatic BP monitor (FT-200S; Jawon Medical, Gyeongsan, Korea) after the patients had rested for 20 min. Participants were instructed to fast for 12 h prior to blood collection. Blood samples were collected in ethylenediaminetetraacetic acid (EDTA)-coated tubes, centrifuged to separate plasma and serum, and then stored at − 70 °C.

### Hematology, biochemical parameters and lipid profile measurements

Hematology, including the white blood cell (WBC) count, red blood cell (RBC) count, hemoglobin levels, hematocrit, platelet count, serum albumin levels, and total bilirubin levels, was measured. For the biochemistry analysis, alanine aminotransferase (ALT), aspartate aminotransferase (AST), alkaline phosphatase (ALP), gamma-glutamyl transferase (GTP), blood glucose, blood urea nitrogen (BUN), creatinine, creatine kinase, lactate dehydrogenase (LDH) activity and high-sensitivity C-reactive protein (hs-CRP) levels were measured. Regarding the lipid profile, total cholesterol, LDL cholesterol, HDL cholesterol, TG, lipoprotein(a) (Lp(a)), free fatty acids, Apo A1, Apo B, and oxidized LDL levels were measured. Detailed descriptions of the methods and complete vendor information are provided in the supplementary file.

### Daily energy intake and physical activity

A 24-h recall method and a semiquantitative food frequency questionnaire were used to acquire data about the subjects’ usual diets. A registered dietitian provided all subjects written and verbal instruction on how to complete a three-day (2 week days and one weekend day) dietary record. Dietary energy estimates and nutrient contents based on 24-h recall were assessed using the Computer-Aided Nutritional analysis program (CAN-pro 3.0, Korean Nutrition Society, Seoul, Rep. of Korea). Total energy expenditure (kcal/day) was calculated from a standardized physical activity record. Basal metabolic rates for each subject were calculated using the Harris-Benedict equation.

### Statistical analysis

All data were analyzed using the IBM SPSS version 24.0 software package (IBM/SPSS, IL, USA). Independent *t*-tests were used to compare parameters between the placebo and test groups. Chi-square tests were used for noncontinuous variables. Paired *t*-tests were used to compare parameters before and after the consumption of the placebo or test product. The results are presented as means ± standard errors.

## Results

This study screened 252 volunteers and initially enrolled 80 subjects. The results from 77 subjects, excluding dropouts, were analyzed. None of the withdrawals were attributed to serious adverse effects. The first participant was enrolled in May 2017, and the last (80th) participant ended the 12-week follow-up in March 2018.

### Basic characteristics and nutritional intake

Table 1 outlines the basic characteristics at baseline and at follow-up for the two groups. Significant differences in the average age, sex ratio, smoking and drinking habits, weight, systolic and diastolic BP, BMI, waist and hip circumference, fat percentage, or lean body mass were not observed between the two groups. Furthermore, significant differences in the basal metabolic rate, total energy expenditure, total energy intake, carbohydrate, protein, fat, total cholesterol and saturated/unsaturated fat intake were not observed between the two groups or within the uRC group and the placebo group over 12 weeks.

**Table 1:** Basic characteristics and nutrition intake values in placebo and test group at baseline and 12-week follow up

	Placebo group (***n*** = 38)		uRC group (***n*** = 39)		***P***^***a***^	***P***^***b***^
	Baseline	Follow-up	Baseline	Follow-up		
Age (year)	47.61	± 1.98	47.03	± 1.97	0.836	
Male/Female n, (%)	6 (15.8) / 32 (84.2)		13 (33.3) / 26 (66.7)		0.074	
Current smoker n, (%)	3 (7.9)		6 (15.4)		0.112	
Current drinker n, (%)	19 (50.0)		20 (51.3)		0.910	
Weight (kg)	61.13 ±1.63	60.93 ±1.68	62.44 ±1.91	62.33 ±1.87	0.605	0.582
Systolic BP (mmHg)	112.09 ±1.92	114.41 ±2.14	113.06 ±2.23	116.97 ±1.77	0.742	0.357
Diastolic BP (mmHg)	69.57 ±1.47	70.92 ±1.64	71.55 ±1.62	72.83 ±1.55	0.368	0.400
Body mass index (kg/m^2^)	23.58 ±0.53	23.50 ±0.54	23.47 ±0.48	23.43 ±0.47	0.873	0.926
Waist hip ratio	0.88 ±0.01	0.88 ±0.01	0.89 ±0.01	0.89 ±0.01	0.257	0.219
Waist circumference (cm)	85.13 ±1.21	85.03 ±1.25	85.92 ±1.29	85.91 ±1.28	0.656	0.622
Hip circumference (cm)	96.96 ±0.96	96.91 ±0.97	96.42 ±0.85	96.37 ±0.84	0.675	0.672
Fat percentage (%)	30.34 ±1.05	30.42 ±1.07	28.21 ±0.95	28.71 ±0.95	0.136	0.235
Lean body mass (kg)	42.49 ±1.29	42.27 ±1.30	44.87 ±1.54	44.51 ±1.54	0.241	0.271
*Estimates of daily nutrient intakes*						
Basal metabolic rate (kcal/day)	1335.81 ±41.80	1331.31 ±42.98	1408.47 ±52.86	1405.88 ±51.97	0.288	0.275
Total energy expenditure (kcal/day)	1926.81 ±42.43	1933.54 ±42.10	2027.71 ±50.36	2033.93 ±51.79	0.132	0.139
Total calorie intake (kcal/day)	1946.27 ±37.67	1944.47 ±40.27	2065.92 ±48.75	2059.22 ±47.44	0.058	0.071
Carbohydrate (%)	61.54 ±0.12	61.67 ±0.13	61.53 ±0.13	61.53 ±0.13	0.992	0.443
Carbohydrate intake (g/day)	299.50 ±5.96	299.64 ±5.97	317.77 ±7.46	316.75 ±7.29	0.060	0.075
Protein (%)	15.83 ±0.06	15.89 ±0.06	15.85 ±0.06	15.82 ±0.06	0.767	0.392
Protein intake (g/day)	77.01 ±1.51	77.30 ±1.67	81.87 ±1.96	81.48 ±1.93	0.054	0.106
Fat (%)	22.64 ±0.15	22.42 ±0.15	22.63 ±0.15	22.66 ±0.15	0.948	0.346
Fat intake (g/day)	48.95 ±0.97	48.51 ±1.16	52.08 ±1.35	51.82 ±1.30	0.067	0.061
Cholesterol intake (mg)	179.30 ±0.40	180.14 ±1.08	180.18 ±0.59	179.63 ±0.36	0.252	0.642
Saturated fatty acid intake (mg)	6.03 ±0.23	6.08 ±0.24	6.51 ±0.27	6.44 ±0.25	0.173	0.301
Monounsaturated fatty acid intake (mg)	10.05 ±0.39	10.08 ±0.39	10.74 ±0.44	10.68 ±0.39	0.245	0.286
Polyunsaturated fatty acid intake (mg)	20.78 ±0.85	20.86 ±0.89	22.57 ±1.01	22.58 ±0.97	0.182	0.199

Mean ± SE (Standard Error), *P*^*a*^-values derived from chi-square test (non-continuous variables) or independent *t*-test at baseline between the placebo and the uRC group. *P*^*b*^-values derived from independent *t*-test at 12-week follow up between the placebo and the uRC group

### Lipid-related parameters

**Fig. 2:**
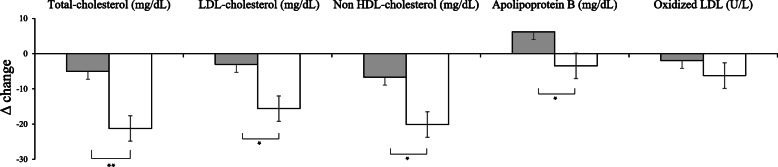
Comparison of the change values of total cholesterol, LDL-cholesterol, non-HDL-cholesterol and apolipoprotein B levels between the placebo (■) group and the uRC (□) group before and after supplementation of uRC. *P < 0.05 and **P < 0.01 derived from independent t-test. A two-tailed *P*-value of less than 0.05 was considered statistically significant. Δ (delta, change) values were calculated as the difference from the baseline (0-week). Data are presented as means Mean ± SE

The changes in lipid profiles from baseline to the 12-week follow-up visit in participants stratified according to the intervention are outlined in Fig. 2 and Table 2. The total cholesterol level was significantly decreased in the uRC group after 12 weeks of supplementation (*P =* 0.006). At the 12-week visit, significantly greater decrease in the total cholesterol level was observed in the uRC group (− 21.23 ± 4.36 mg/dL) than in the placebo group (− 5.05 ± 3.84 mg/dL; *P =* 0.007). A significantly greater decrease in the LDL cholesterol level was observed in the uRC group (− 15.61 ± 4.16 mg/dL) than in the placebo group (− 3.05 ± 3.95 mg/dL; *P =* 0.032). Non-HDL cholesterol levels were significantly reduced in the uRC group after 12 weeks of supplementation (− 20.13 ± 4.14 mg/dL) compared with the placebo group (− 6.68 ± 3.30 mg/dL; *P =* 0.013). At the 12-week visit, the reduction in Apo B levels was significantly greater in the uRC group (− 3.48 ± 3.40 mg/dL), but Apo B levels were increased in the placebo group (6.21 ± 2.84 mg/dL; *P =* 0.032). In addition, a remarkably lower oxidized LDL level was observed in the uRC group (57.76 ± 2.07 U/L) than in the placebo group (66.09 ± 3.47 U/L) after 12 weeks of supplementation (*P =* 0.044). Interestingly, no significant differences were observed in TG, HDL cholesterol, free fatty acid, Apo A1, or Lp(a) levels. Detailed information on the five nonsignificant markers (Supplementary Table 1) and the relationships among changes in the levels of total cholesterol, non-HDL cholesterol, LDL cholesterol, Apo B, and oxidized LDL are shown in the supplementary data (Supplementary Fig. 1).

**Table 2:** Lipid-related parameters of placebo and test group at baseline and 12-week follow up

	Placebo group (***n*** = 38)				uRC group (***n*** = 39)				***P***^***a***^	***P***^***b***^	***P***^***c***^
	Baseline		Follow-up		Baseline		Follow-up				
Total-cholesterol (mg/dL)	213.86	±1.79	208.81	±3.95	214.05	±1.50	192.82	±4.07	0.938	0.006	
*Change*		*−5.05*	*±3.84*			*−21.23*	*±4.36*				*0.007*
Triglyceride (mg/dL)	111.45	±6.22	119.26	±7.70	116.05	±7.13	115.49	±8.13	0.629	0.737	
*Change*		*−7.82*	*±7.84*			*--0.56*	*±7.06*				*0.429*
LDL-cholesterol (mg/dL)	135.60	±2.58	132.55	±4.26	133.41	±2.06	117.79	±4.34	0.508	0.018	
*Change*		*−3.05*	*±3.95*			*−15.61*	*±4.16*				*0.032*
Non-HDL-cholesterol (mg/dL)	153.71	±3.13	147.03	±4.33	153.41	±2.63	133.28	±4.40	0.942	0.029	
*Change*		*−6.68*	*±3.30*			*−20.13*	*±4.14*				*0.013*
Apolipoprotein B (mg/dL)	114.39	±2.75	120.60	±3.82	115.17	±2.83	111.69	±3.46	0.843	0.088	
*Change*		*6.21*	*±2.84*			*−3.48*	*±3.40*				*0.032*
Oxidized LDL (U/L)	68.05	±3.02	66.09	±3.47	64.31	±2.53	57.76	±2.07	0.348	0.044	
*Change*		*−1.95*	*±2.41*			*−6.23*	*±1.96*				*0.174*

Mean ± SE (Standard Error), *P*^*a*^-values derived from independent *t*-test at baseline baseline between the placebo and the uRC group. *P*^*b*^-values derived from independent *t*-test at 12-week follow up between the placebo and the uRC group. *P*^*c*^-values derived from independent *t*-test for changed value between the placebo and the uRC group. Change value (Δ, delta) represents the change from baseline at follow-up

### Hematology and biochemical analysis

The analysis of the biochemical data and levels of liver- and kidney-related biochemical markers, such as AST, ALT, creatinine and BUN, revealed no abnormal findings and did not show any significant differences between groups. In addition, the average hs-CRP level in the uRC group was normal (Supplementary Table 2 and Supplementary Fig. 2).

## Discussion

According to this RCT, dietary supplementation with uRC reduced total blood cholesterol and LDL cholesterol levels in subjects with borderline-high cholesterol levels ranging from 200 to 239 mg/dL. This finding has important clinical implications because elevated LDL cholesterol levels account for approximately half of the risk of myocardial infarction [4] and approximately a quarter of the risk of ischemic stroke [35]. Additionally, uRC supplementation improved the lipid profile, including a reduction in non-HDL cholesterol, Apo B and oxidized LDL levels.

Although the effect of uRC on lipid profiles in individuals with metabolic syndrome has been reported [25], the effect of uRC on subjects with borderline-high total cholesterol levels has not been studied*.* In the study by Myung et al., uRC supplementation improved vascular endothelial function in 51 statin-naïve participants with metabolic syndrome during a 12-week follow-up [25]. In this previous study, a greater reduction in total cholesterol levels from baseline (− 22.7 ± 34.3 mg/dL vs. 0.0 ± 34.7 mg/dL, *P* < 0.05) was observed in the supplementation group than in the placebo group.

An intervention with a functional food or nutraceutical product is recognized to improve lipid profiles [36, 37]. Some possible mechanisms of the interaction between functional food intervention and blood lipid levels have been identified, including decreased lipid peroxidation [37], improved superoxide dismutase activity [38], antioxidant enzyme stimulation [39, 40], reduced oxidative stress and LDL oxidation [41, 42]. Additionally, the consumption of high-phenolic products attenuates atherosclerosis by increasing the antioxidant capacity [43–45]. In particular, uRC has been reported to activate or induce the overexpression of glutathione peroxidase, which is an endogenous catalytic H_2_O_2_ scavenger, and this property may inhibit the development of atherosclerosis [46].

uRC exerts a cholesterol-lowering effect due to its functionally active compounds. According to Choi et al. [20], the components of uRC were analyzed as physiologically active substances. Ellagic acid was reported to be the most abundant ingredient, and the ellagic acid content of unripe fruit was approximately seven-fold higher than ripe fruit [47]. Park et al. reported that ellagic acid promotes cholesterol efflux by exerting inhibitory effects on lipid accumulation and scavenger receptor class B type 1 induction [48]. Additionally, by reducing lectin-like oxidized LDL receptor-1-mediated signaling, ellagic acid suppresses endothelial dysfunction [47]. Another study reported the anti-lipid peroxidation properties and antihyperlipidemic activity mediated by the inhibition of β-hydroxy β-methylglutaryl-CoA (HMG-CoA) reductase by ellagic acid [49]. In this experimental study, Kannan et al. postulated that the high affinity of ellagic acid for the lipid membrane of the myocardium might be an important factor contributing its antihyperlipidemic effect.

Previous studies reported the effects of uRC on improving the lipid profile [23, 50]. In preclinical studies conducted by the authors, uRC was confirmed to inhibit lipid accumulation in 3 T3-L1 adipocytes in a dose-dependent manner [31]. In that study, oral administration of the uRC (50 and 100 mg/kg/day) extract for 12 weeks resulted in an impressive reduction in total cholesterol and LDL cholesterol levels in obese mice. In vivo, uRC extracts decreased the levels of the peroxisome proliferator-activated receptor γ (PPARγ) mRNA in adipose tissues. In addition, in 2015, five bioactive isolated compounds (ellagic acid, erycibelline, 5-hydroxy-2-pyridinemethanol, m-hydroxyphenylglycine, and 4-hydroxycoumarin) were investigated and shown to decrease lipid accumulation in 3 T3-L1 cells. Moreover, the expression of the CCATT/enhancer-binding protein α (C/EBPα), SREBP-1c, acetyl-CoA carboxylase (ACC), and fatty acid synthase (FAS) mRNAs was decreased [32].

Most recently, Lee et al. confirmed the hypocholesterolemic mechanism of its major bioactive compound, ellagic acid, in vitro and in vivo (hepatocyte and high-cholesterol diet-induced rat model) [51]. After the administration of uRC extract, the hepatic activity of lecithin cholesterol acyltransferase increased and the serum malondialdehyde content decreased. Furthermore, immunoblot assays showed increased 5′ adenosine monophosphate-activated protein kinase (AMPK) phosphorylation in the liver and decreased cholesterol biosynthesis because sterol regulatory element binding protein-2 (SREBP-2) activation was inhibited by uRC administration.

In addition to ellagic acid, uRC contains high levels of tyrosol, resveratrol, ascorbic acid, anthocyanin, flavonoids and phenolic acid [34, 52, 53]. These physiologically active components exert antioxidant and antiatherosclerotic effects by inhibiting the formation of inducible nitric oxide synthase (iNOS) and endothelial nitric oxide synthase (eNOS) [21–24]. Tyrosol and resveratrol decrease the uptake of oxidized LDL by monocytes/macrophages through the modulation of the c-Jun N-terminal kinase signaling pathway [54, 55]. Ascorbic acid and anthocyanin are abundant components among the phenolic compounds and inhibit free radical activity, exerting significant antioxidant effects [53]. Furthermore, anthocyanin also exerts anti-inflammatory effects, prevents the oxidation of LDL cholesterol, and inhibits platelet aggregation. Vasodilatation by nitrate oxide has also been reported in coronary arteries [56]. The flavonoids present in uRC increase vascular expansion by endothelial cells in patients with vascular endothelial dysfunction [57]. A likely explanation for this finding is that flavonoids increase the bioavailability of NOS in the vascular endothelium [58, 59]. Additionally, numerous studies have reported the protective effect of polyphenols on LDL oxidation [60–62]. Polyphenolic compounds increase nitric oxide production and the accumulation of cyclic guanosine monophosphate (cGMP) in the vascular endothelium [63]. Thus, supplementation with uRC potentially exerts antihyperlipidemic effects that are mediated by the pharmaceutical functions of its active components.

Apo B, a predictor of ischemic cardiovascular events [64], can measure the total number of lipogenic particles in plasma. As shown in the study by Benn et al. [64], Apo B levels in the upper tertile confer higher risks of ischemic heart disease, myocardial infarction, and any ischemic cardiovascular event than Apo B levels in the lower tertile, with hazard ratios ranging from 1.6 to 2. Therefore, the beneficial effect of uRC supplementation on Apo B and LDL cholesterol levels observed in this study might be related to the prevention of cardiovascular events.

Because oxidized LDL functions as a proinflammatory factor in atherosclerosis [48], inhibition of the uptake of oxidized LDL represents a major therapeutic target for atherosclerosis [65]. Briefly, LDL accumulates within the intimal space and undergoes oxidation to form oxidized LDL [66]. In this regard, the decreased circulating Apo B and oxidized LDL levels observed in the current study may provide valuable insights into the antiatherosclerotic efficacy of the uRC supplement.

Lp(a) is known to possess atherogenic properties [67, 68]; moreover, increased Lp(a) levels are associated with the incidence of atherosclerosis [69, 70] and a higher CVD risk [71]. Furthermore, the European Atherosclerosis Society and the National Lipid Association guidelines recommend monitoring Lp(a) levels in patients at high risk of CVD [72, 73]. Borderline-high cholesterol levels can precede dyslipidemia and CVD, and Lp(a) is potentially an atherogenic parameter; thus, Lp(a) levels were measured. However, although total cholesterol, LDL cholesterol, oxidized LDL and Apo B levels were significantly improved, a significant difference in Lp(a) levels was not observed between the two groups. A potential explanation for this result is that Lp(a) levels are highly heterogeneous between various individuals [74], and the Lp(a) level is less likely to be affected by dietary changes, although LDL and Apo B-100 levels are altered by interventions [69, 75]. Additionally, even though Lp(a) shares a common structure with LDL, the distinct metabolic mechanisms regulating cholesterol and Lp(a) levels might contribute to the nonsignificant result observed after uRC supplementation [70].

Although a significant difference was not observed due to the sample size, the average hs-CRP level in the uRC group (0.70 ± 5.36 to 1.75 ± 3.69 mg/L) showed an increasing trend compared with the placebo group, and this tendency differed from the reductions in LDL cholesterol (133.41 ± 2.06 to 117.79 ± 4.34 mg/dL) and oxidized LDL (64.31 ± 2.53 to 57.76 ± 2.07 U/L) levels in the uRC group. However, because all participants were at high risk of hypercholesterolemia, we were unable to easily determine whether the increased hs-CRP level and loss of relation to changes in LDL cholesterol or oxidized LDL levels are abnormal reactions. Duarte et al. [76] measured hs-CRP levels in subjects with hypercholesterolemia (*n* = 37); the average hs-CRP level of the test group was 20.28 ± 3.05 mg/L, while the level of the normal group was 1.80 ± 0.11 mg/L. Additionally, 14 (37.8%) subjects presented with high hs-CRP values exceeding 20 mg/L in the hypercholesterolemic group. Furthermore, given the wide range of hs-CRP levels in the small group analyzed in the present study, as shown in the supplementary figure, a significant positive correlation between LDL cholesterol and oxidized LDL and hs-CRP levels was not observed.

In addition, the HDL cholesterol level did not increase and did not show a statistically significant difference. A potential explanation for this finding is that the HDL cholesterol levels in both groups at baseline were within the normal and recommended range [5–7]. As a result, an improvement in HDL cholesterol levels was not observed in the before-and-after comparison of the administration of uRC.

### Study strengths and limitations

To the authors’ knowledge, this study is the first double-blinded RCT to examine blood lipid profile levels after uRC intervention in human borderline-high cholesterol subjects. This RCT study possesses several study strengths; it elucidates the efficacy of uRC in LDL cholesterol improvement as the primary outcome and reports variety and sufficient human clinical data including study background and randomization design, test product manufacturing and dosage information, inclusion and exclusion criteria, anthropometric, dietary, hematologic, biochemical results and other sub-analyzed supplementary data. Also, this study could resolve safety concerns about uRC consumption because there were no reports of adverse effects nor abnormal findings in liver- and kidney-related markers during the 12-week intervention in 80 subjects.

Limitations of the current study include the relatively short duration of the single-center study. Additionally, further evaluations, such as artery pulse wave velocity or intima-media wall thickness and subfractions of lipoproteins, were not conducted. The uncertain result of hs-CRP levels, which might be due to the small sample size and selection of the subjects, was another limitation. Moreover, the lack of analysis of a dose-response relationship between uRC intake and changes in the lipid profile of human subjects is also a limitation of the present study. Nevertheless, uRC supplementation reduces LDL cholesterol, non-HDL cholesterol, Apo B and oxidized LDL levels compared with a placebo supplement among patients with borderline-high total cholesterol levels. To strengthen evidence for patient care or initiate a uRC mixed-drug discovery process, clinical trials are still required with a combination of a cholesterol-lowering drug treatment and uRC supplementation, examinations of various dosages of uRC compared with other antiatherosclerotic agents or lifestyle modifications, investigations exploring disease-specific mechanisms and patient-specific effects of uRC, and evaluation of the synergetic interaction between uRC and other functional bioactive components.

## Conclusions

This study can provide an in-depth understanding of further uses of uRC and the broad potential implications of unhealthy lipid-related conditions. Since uRC is naturally rich in ellagic acids and other polyphenolic elements, supplementation with uRC improves lipid biomarker status. In conclusion, these new findings confirm the scientific underpinnings of uRC’s cholesterol-lowering effect. This strict and well-designed clinical trial indicates that uRC has considerable future prospects as a highly promising therapeutic agent and can be used to treat lipid disorder patients. Preventively and/or therapeutically, together with other remedies or alone, uRC has potential development opportunities in lipidologic therapy.

## Supplementary information

**Additional file 1: Supplementary Table 1.** Lipid-related parameters of placebo and test group at baseline and 12-week follow up. **Supplementary Table 2.** Hematology and biochemical parameters of placebo and test group at baseline and 12-week follow up. **Supplementary Figure 1.** Relationship among changed levels of total cholesterol, non-HDL-cholesterol, LDL-cholesterol and apolipoprotein B, and oxidized-LDL. **Supplementary Figure 2.** Relationship and distribution of hs-CRP, LDL cholesterol and oxidized LDL levels in each group at baseline and follow-up.

## Data Availability

Not applicable.

## Abbreviations

ACS-ACOD: Acyl-CoA synthetase-acyl-CoA oxidase
ALP: Alkaline phosphatase
ALT: Alanine aminotransferase
AMPK: Adenosine monophosphate-activated protein kinase
Apo: Apolipoprotein
AST: Aspartate aminotransferase
BMI: Body mass index
BP: Blood pressure
BUN: Blood urea nitrogen
cGMP: Cyclic guanosine monophosphate
EDTA: Ethylenediaminetetraacetic acid
GMP: Good manufacturing practice
GOT: Glutamic oxaloacetic transaminase
GPT: Glutamic pyruvic transaminase
HDL: High-density lipoprotein
HFF: Health functional food
HMG-CoA: β-hydroxy β-methylglutaryl-CoA
HPLC: High-performance liquid chromatography
INDELs: Insertions and deletions
KFDA: Korea Food and Drug Administration
LDH: Lactate dehydrogenase
LDL: Low-density lipoprotein
Lp (a): Lipoprotein (a)
LPS: Lipopolysaccharide
NCEP: National Cholesterol Education Program
NO: Nitric oxide
NOS: Inducible nitric oxide synthase
PGE2: Prostaglandin E2
RC: *Rubus coreanus*
RCT: Randomized double-blind controlled trial
SNP: Single nucleotide polymorphism
TG: Triglyceride
uRC: Unripe *Rubus coreanus*
VLDL: Very low-density lipoprotein
