# Supplementary material for: The cholesterol-lowering effect of unripe Rubus coreanus is associated with decreased oxidized LDL and apolipoprotein B levels in subjects with borderline-high cholesterol levels: a randomized controlled trial
Source: Lipids Health Dis. 2020 Jul 9;19:166. doi: 10.1186/s12944-020-01338-z (PMC7350759; doi:10.1186/s12944-020-01338-z)
Supplement: Supplementary file 1 — Additional file 1: Supplementary Table 1. Lipid-related parameters of placebo and test group at baseline and 12-week follow up. Supplementary Table 2. Hematology and biochemical parameters of placebo and test group at baseline and 12-week follow up. Supplementary Figure 1. Relationship among changed levels of total cholesterol, non-HDL-cholesterol, LDL-cholesterol and apolipoprotein B, and oxidized-LDL. Supplementary Figure 2. Relationship and distribution of hs-CRP, LDL cholesterol and oxidized LDL levels in each group at baseline and follow-up. [file 12944_2020_1338_MOESM1_ESM.doc]

**Supplementary Table 1. Lipid-related parameters of placebo and test group at baseline and 12-week follow up.**

|  | **Placebo group (*n*=38)** | | | | **uRC group (*n*=39)** | | | | ***P^a^*** | ***P^b^*** |
| --- | --- | --- | --- | --- | --- | --- | --- | --- | --- | --- |
|  | **Baseline** | | **Follow-up** | | **Baseline** | | **Follow-up** | |  |  |
| HDL-cholesterol (mg/dL) | 60.15 | ±2.96 | 61.78 | ±2.83 | 60.64 | ±2.73 | 59.53 | ±2.65 | 0.905 | 0.564 |
| Apolipoprotein A1 (mg/dL) | 195.94 | ±5.63 | 195.78 | ±5.65 | 202.00 | ±5.66 | 195.61 | ±5.59 | 0.451 | 0.938 |
| Free fatty acid (uEq/L) | 633.47 | ±56.2 | 635.78 | ±46.4 | 801.3 | 3±69.2 | 676.28 | ±70.2 | 0.064 | 0.631 |
| Lipoprotein (a) (mg/dL) | 11.17 | ±1.05 | 12.91 | ±1.38 | 10.83 | ±1.02 | 12.68 | ±1.17 | 0.818 | 0.898 |

Mean ± SE (Standard Error), *P^a^*-values derived from independent *t*-test at baseline. *P^b^*-values derived from independent *t*-test at 12-week follow up.

**Supplementary Table 2. Hematology and biochemical parameters of placebo and test group at baseline and 12 week follow up.**

|  | **Placebo group (*n*=38)** | | | | | | **uRC group (*n*=39)** | | | | | | ***P^a^*** | | ***P^b^*** | |
| --- | --- | --- | --- | --- | --- | --- | --- | --- | --- | --- | --- | --- | --- | --- | --- | --- |
|  | **Baseline** | | | **Follow-up** | | | **Baseline** | | | **Follow-up** | | |  |  |  |  |
| White Blood Cell (ｘ10^3^/μL) | 5.14 | ±0.22 | 5.49 | | ±0.22 | 5.62 | | ±0.23 | 5.21 | | ±0.21 | 0.136 | | 0.364 | |  |
| Red Blood Cell (ｘ10^6^/mm^3^) | 4.54 | ±0.08 | 4.63 | | ±0.09 | 4.69 | | ±0.10 | 4.73 | | ±0.07 | 0.423 | | 0.762 | |  |
| Hemoglobin (g/dL) | 13.60 | ±0.22 | 14.06 | | ±0.31 | 14.06 | | ±0.25 | 14.18 | | ±0.23 | 0.222 | | 0.722 | |  |
| Hematocrit (%) | 41.02 | ±0.63 | 42.31 | | ±0.77 | 41.91 | | ±0.92 | 42.85 | | ±0.70 | 0.431 | | 0.602 | |  |
| Platelet (ｘ10^3^/mm^3^) | 227.51 | ±6.69 | 228.21 | | ±7.82 | 222.76 | | ±7.15 | 226.53 | | ±6.02 | 0.629 | | 0.865 | |  |
| Serum albumin (g/dL) | 4.67 | ±0.06 | 4.70 | | ±0.06 | 4.63 | | ±0.05 | 4.60 | | ±0.05 | 0.782 | | 0.694 | |  |
| AST (IU/L) | 22.34 | ±0.82 | 23.18 | | ±0.93 | 23.58 | | ±1.20 | 23.05 | | ±1.03 | 0.406 | | 0.924 | |  |
| ALT (IU/L) | 17.53 | ±1.36 | 19.24 | | ±1.88 | 20.18 | | ±1.63 | 19.90 | | ±1.63 | 0.217 | | 0.791 | |  |
| γ-GTP (U/L) | 19.76 | ±2.97 | 19.55 | | ±2.89 | 30.69 | | ±9.00 | 25.21 | | ±5.09 | 0.257 | | 0.341 | |  |
| BUN (mg/dL) | 14.00 | ±0.52 | 14.34 | | ±0.51 | 14.95 | | ±0.57 | 14.31 | | ±0.56 | 0.224 | | 0.964 | |  |
| Creatinine (mg/dL) | 0.74 | ±0.02 | 0.70 | | ±0.02 | 0.76 | | ±0.03 | 0.70 | | ±0.03 | 0.547 | | 0.920 | |  |
| Total bilirubin (mg/dL) | 0.66 | ±0.03 | 0.66 | | ±0.04 | 0.66 | | ±0.04 | 0.62 | | ±0.03 | 0.966 | | 0.330 | |  |
| ALP (U/L) | 60.16 | ±2.54 | 59.18 | | ±2.55 | 57.64 | | ±2.80 | 57.03 | | ±2.74 | 0.508 | | 0.567 | |  |
| Total protein (g/dL) | 7.24 | ±0.10 | 7.24 | | ±0.08 | 7.24 | | ±0.11 | 7.13 | | ±0.09 | 0.994 | | 0.370 | |  |
| Creatine kinase (IU/L) | 113.08 | ±12.8 | 106.21 | | ±26.4 | 117.82 | | ±12.7 | 89.00 | | ±7.30 | 0.793 | | 0.528 | |  |
| Lactate dehydrogenase (IU/L) | 174.97 | ±3.88 | 175.53 | | ±4.36 | 182.15 | | ±5.36 | 172.05 | | ±3.69 | 0.283 | | 0.544 | |  |
| hs-CRP (mg/L) | 1.39 | ±0.53 | 1.12 | | ±0.32 | 0.70 | | ±5.36 | 1.75 | | ±3.69 | 0.215 | | 0.368 | |  |

Mean ± SE (Standard Error), *P^a^*-values derived from independent *t*-test at baseline. *P^b^*-values derived from independent *t*-test at 12-week follow up.

Note: No significant differences and abnormal findings were found in serum concentrations of white blood cells, red blood cells, hemoglobin, hematocrit, platelets, serum albumin, GOT, GPT, γ-GTP, BUN, creatinine, total bilirubin, ALP, total protein, creatine kinase, LDH or hs-CRP at baseline between the placebo and test groups.

<**Blood sample measurement methodologies>**

**Hematology and biochemical parameters**

Hematology, including white blood cell (WBC) counts, red blood cell (RBC) counts, hemoglobin levels, hematocrit, platelet, serum albumin levels, total bilirubin levels, was analyzed using an automated blood counting analyzer (HORIBA Ltd., Tokyo, Japan). Total bilirubin levels were measured using the vanadate oxidation method with a HITACHI 7600 automatic analyzer (HITACHI Ltd., Tokyo, Japan). Alanine aminotransferase (ALT), aspartate aminotransferase (AST), and alkaline phosphatase (ALP) levels were measured using the IFCC UV method with a HITACHI 7600 automatic analyzer. Gamma-glutamyl transferase (GTP) activity was assessed by measuring the change in the absorbance of 5-amino-2-nitrobenzoic acid produced by the enzymatic reaction. Blood glucose levels were measured in venous blood samples collected from patients after a 12-h fast using the hexokinase method with a HITACHI 7600 automatic analyzer. Total protein concentrations were measured with the Biuret method using a Cobas 8000 c702 automatic analyzer (Roche Ltd., Basel, Switzerland). Blood urea nitrogen (BUN) levels were measured using a HITACHI 7600 automatic analyzer with a kinetic UV assay for urea and urea nitrogen levels. Creatinine levels were measured by measuring the concentration of the reaction product with picric acid in an alkaline solution at a wavelength of 505 nm using a HITACHI 7600 automatic analyzer. Creatine kinase and lactate dehydrogenase (LDH) activity were measured with a colorimetric assay at a wavelength of 340 nm using a HITACHI 7600 automatic analyzer.

**Lipid profile measurements**

Serum total cholesterol and triglyceride levels were measured with an enzymatic method using an Auto Chemistry Analyzer Express Plus (Chiron Diagnostics Co., MA, USA). LDL-cholesterol levels were measured using a HITACHI 7600 automatic analyzer with an enzymatic method using the LDL-C plus (Roche Ltd., Mannheim, Germany) reagent. In the presence of high-density lipoprotein (HDL) cholesterol, dextran sulfate selectively forms a water-soluble complex that is resistant to LDL, very low-density lipoprotein (VLDL), chylomicrons and purified lipoprotein lipase (PEG-modified) enzymes in the presence of magnesium ions. HDL cholesterol levels were measured with a homogeneous enzymatic colorimetric method using HDL-C plus Gen.3 (Roche Ltd.). Lipoprotein(a) (Lp(a)) levels were assessed with the turbidity immunoassay method using an Lp(a) Latex (SEKISUI, Osaka, Japan) reagent and measured using a HITACHI 7600 automatic analyzer. Free fatty acid levels were measured using a Hitachi 7600 automatic analyzer based on the principle of the acyl-CoA synthetase-acyl-CoA oxidase (ACS-ACOD) method. Apolipoprotein A1 and apolipoprotein B levels were analyzed using an immunoturbidimetric method. Plasma oxidized-LDL levels were measured using an enzyme immunoassay (Mercodia AB, Uppsala, Sweden), and the resulting color of the reaction was monitored at 450 nm with a Wallac 1420 Victor2 multilabel counter (PerkinElmer Life Sciences, MA, USA).


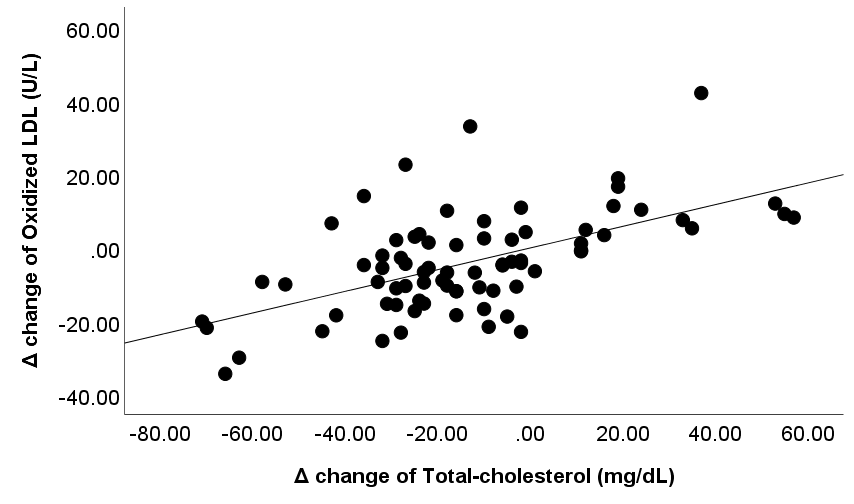

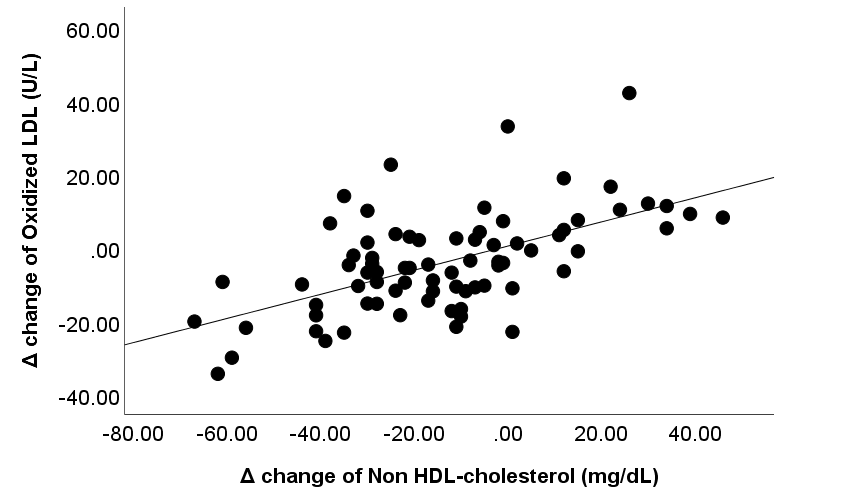

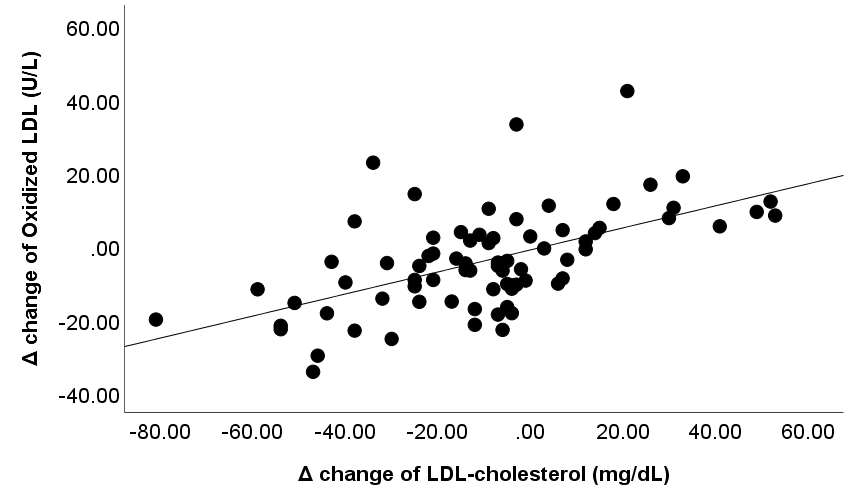

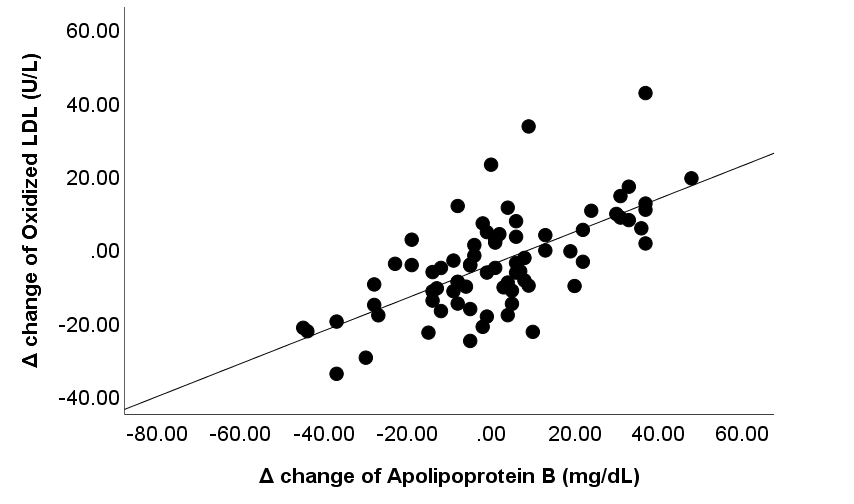


**Supplementary Figure 1. Relationship among changed levels of total cholesterol, non-HDL-cholesterol, LDL-cholesterol and apolipoprotein B, and oxidized-LDL.**

*r* :Pearson correlation coefficient. Δ (delta) values were calculated as the difference between the baseline and 12-week.

Note: As shown in Figure, net changes of oxidized-LDL (U/L) were positively correlated with those of total cholesterol (r=0.578, *P*<0.001), non-HDL-cholesterol (r=0.584, *P* <0.001), LDL-cholesterol (r=0.271, *P* <0.001) and apolipoprotein B (r=0.660, *P* <0.001).

| Placebo (contorol) group (*n*=38) | Test (uRC) group (*n*=39) |
| --- | --- |
| Baseline (0 week) | |
| 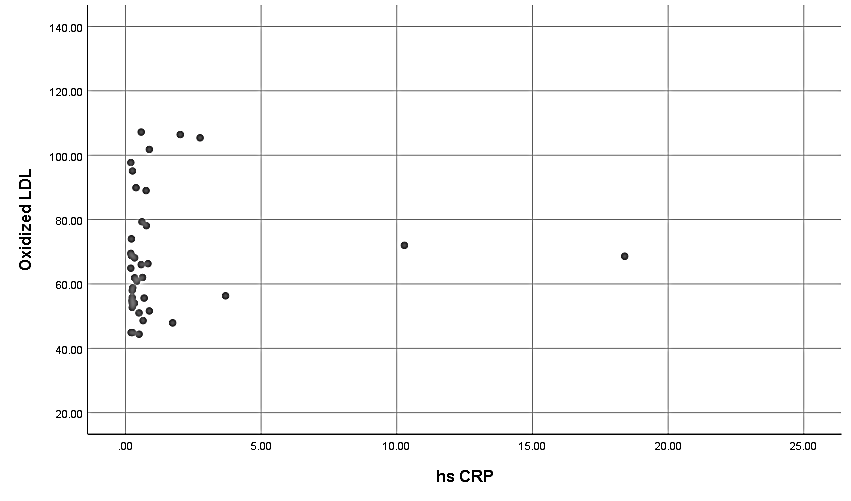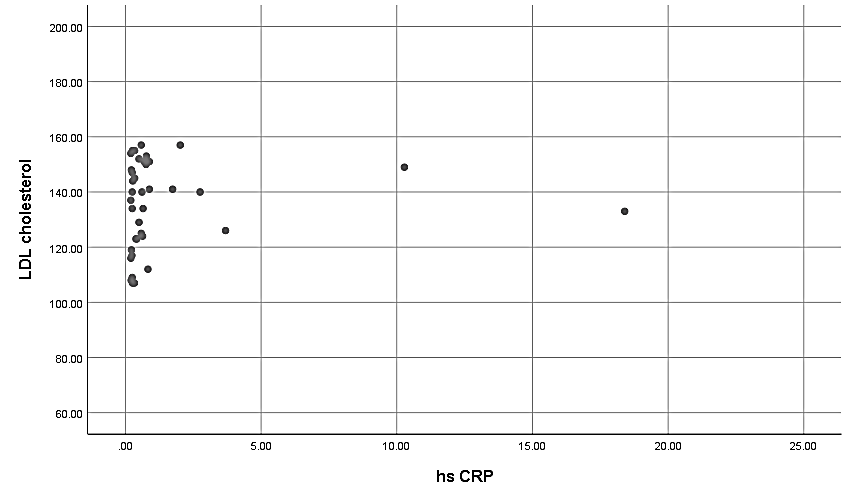 | 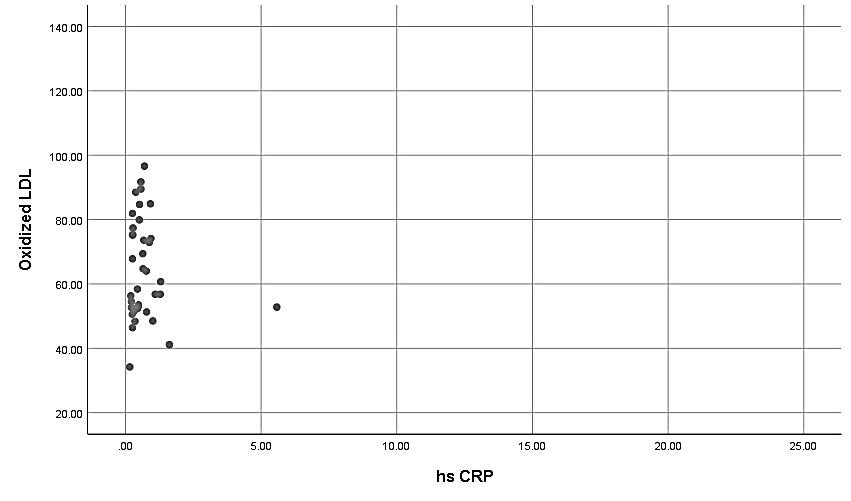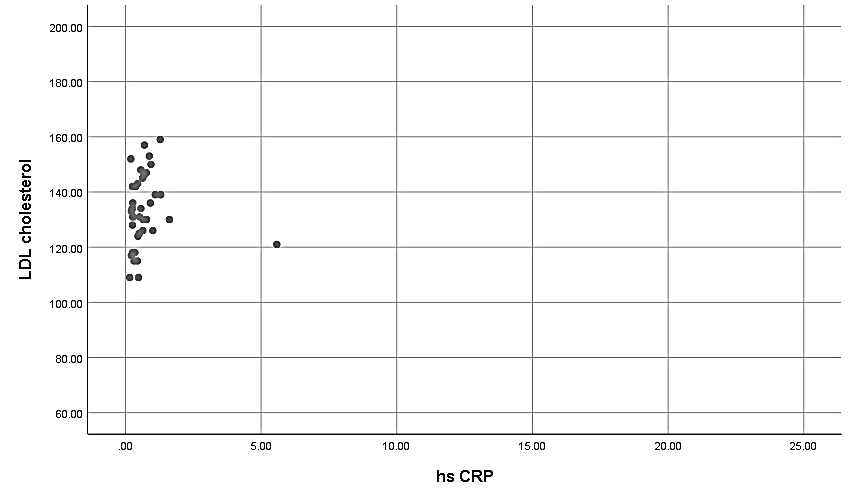 |
| Follow up (12 week) | |
| 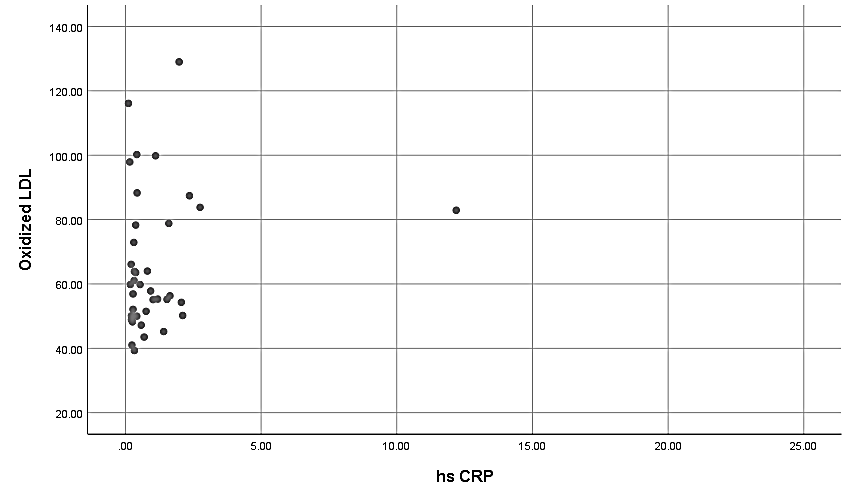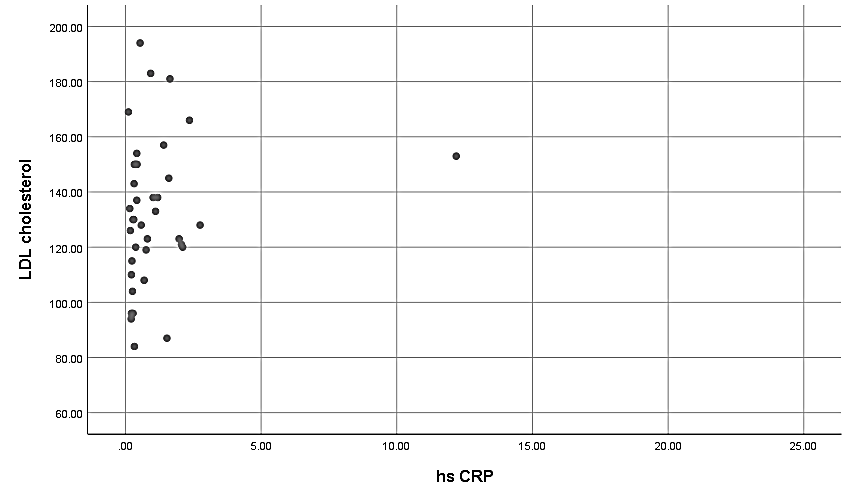 | 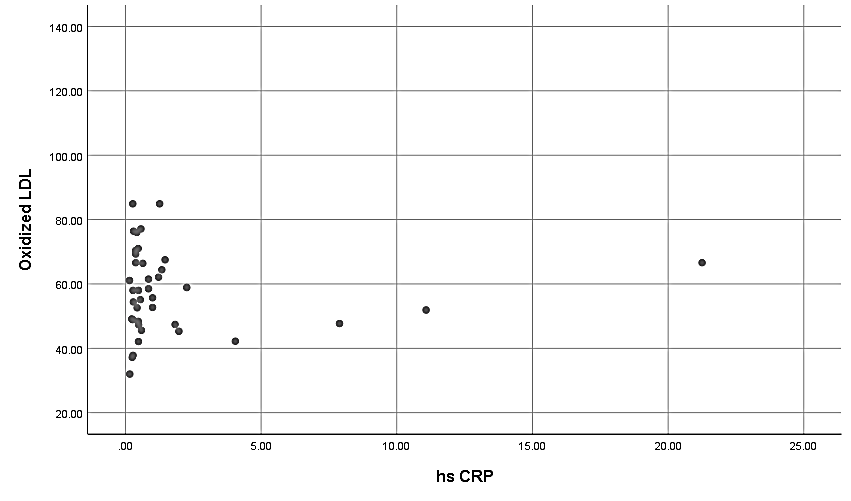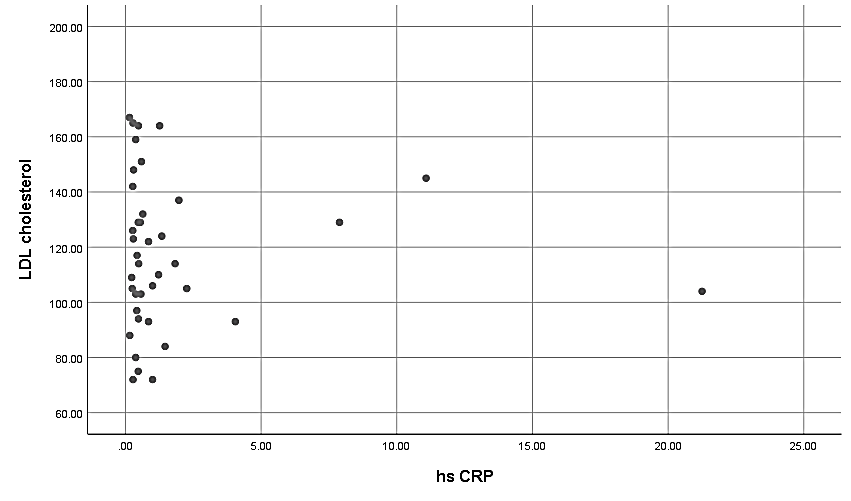 |

**Supplementary Figure 2. Relationship and distribution of hs-CRP, LDL cholesterol and oxidized LDL levels in each group at baseline and follow-up.**
